# Diagnostic Performance of Positron Emission Tomography/Computed Tomography Using Fluorine-18 Fluorodeoxyglucose in Detecting Locoregional Nodal Involvement in Patients with Anal Canal Cancer: A Systematic Review and Meta-Analysis

**DOI:** 10.1155/2014/196068

**Published:** 2014-02-04

**Authors:** Carmelo Caldarella, Salvatore Annunziata, Giorgio Treglia, Ramin Sadeghi, Narjes Ayati, Luca Giovanella

**Affiliations:** ^1^Institute of Nuclear Medicine, Department of Radiological Sciences, Università Cattolica del Sacro Cuore, Rome, Italy; ^2^Institute of Nuclear Medicine, Department of Bioimaging and Radiological Sciences, Università Cattolica del Sacro Cuore, Largo Agostino Gemelli 8, 00168 Rome, Italy; ^3^Department of Nuclear Medicine and PET/CT Center, Oncology Institute of Southern Switzerland, Bellinzona, Switzerland; ^4^Nuclear Medicine Research Center, Mashhad University of Medical Sciences, Mashhad, Iran

## Abstract

*Purpose*. The diagnostic performance of positron emission tomography using ^18^F-fluorodeoxyglucose (FDG-PET) in detecting nodal involvement in patients with anal canal cancer (ACC) has been investigated by several studies with conflicting results. The aim of our study is to systematically review and meta-analyze published data about this topic. *Methods*. A comprehensive computer literature search of PubMed/MEDLINE, Scopus, and Embase databases was carried out on July 10 to find relevant articles concerning the diagnostic performance of FDG-PET in detecting locoregional nodal involvement in patients with ACC. No language restriction was used. Pooled diagnostic performance on a lesion-based analysis was calculated. *Results*. Seven retrospective and five prospective studies have been reviewed. Six studies allowed assessing pooled sensitivity; five studies allowed assessing pooled specificity. Sensitivity and specificity values of FDG-PET/CT on a lesion-based analysis ranged from 31 to 100% and from 53 to 98%, with pooled estimates of 56% (95% CI: 45–67%) and 90% (95% CI: 86–93%), respectively. *Conclusions*. Our meta-analysis demonstrates that FDG-PET is a specific diagnostic tool in detecting locoregional lymph node involvement in patients with ACC. Low sensitivity is a major concern; however, higher sensitivity could be reached combining FDG-PET with MR scan.

## 1. Introduction

Anal canal cancer (ACC) is a relatively uncommon neoplasm, with an incidence of about 1–3 cases per 100,000 and accounting for about 2% of gastrointestinal malignancies [1–3]. Prevalence is about 3-fold higher in women than in men, especially at older ages (5th and 6th decades). Cigarette smoking, receptive anal sex, lifetime number of sexual partners, pelvic radiation therapy, history of genital warts and/or previous neoplasms related to HPV infection, and immunosuppression conditions (transplantation HIV infection) are common risk factors. Particularly, ACC incidence rates are significantly higher in HIV-infected men and women than in HIV-uninfected ones, especially when considering men with homosexual habits [4–6].

Rectal bleeding, usually of small entity, eventually accompanied with anal itching, tenesmus, or change in bowel habits, is the most frequently occurring sign; swollen inguinal or perineal lymph nodes, mucous anal leakage, and ribbon-like stools are far less frequently observed. Fecal incontinence and frank pain while seated are common in locally advanced ACC. However, such signs and symptoms of ACC are not specific for this disease, since more common benign conditions, like hemorrhoids, rectal solitary ulcer, or anal fissure, may arise the same way. Moreover, ACC may be completely asymptomatic in about 20% of patients and incidentally discovered during rectal exam for prostate screening colonoscopy in patients at risk for colon-rectum cancer or even during surgical intervention for hemorrhoids.

Early disease detection and treatment may have a significant positive impact on the overall survival of patients with ACC [7]. Histological diagnosis is mandatory, but the evaluation of the extent of disease is the major concern because treatment plans are quite different in patients with extensive disease rather than in patients with localized ACC. At the time of diagnosis, localized disease (stages I and II) is found in about 50% of patients, while about 30% of patients have locoregional lymph node involvement and 8–12% have evidence of distant metastases [8].

Currently, magnetic resonance imaging (MRI) performed on high-magnetic-field scanners is the imaging modality of choice to investigate the anal region for both staging and treatment evaluation purposes, due to its high-detail visualization of the anal canal and nearby anatomical structures, reliable soft tissue differentiation, noninvasiveness, and not needing special patient preparation [9–11]. Extramural neoplastic spread and infiltration of adjacent organs and sphincter muscle complexes are properly evaluated. MRI may be helpful in evaluating perilesional lymph node involvement: short-axis threshold values for neighboring lymph nodes have been suggested to reliably discriminate between pathological and normal ones; however, morphological (abnormal shape, loss of nodal hilum, and signal heterogeneity) and contrast-enhancement features (inhomogeneous enhancement), in addition to the mere size criterion, may increase specificity in this setting [10, 12].

Computed tomography (CT) performed with multi-detector technique allows a good visualization of the primary lesion, although with a lower contrast resolution than MRI, being a reliable alternative in patients with contraindications to MRI. Nevertheless, CT may be helpful in detecting eventual spread of neoplastic cells in the liver or other organs: indeed, dissemination, though uncommon, is associated with a higher risk of recurrent disease after treatment [13].

The role of positron emission tomography/computed tomography (PET/CT) using fluorine-18 fluorodeoxyglucose (FDG) in staging, response evaluation, and followup of ACC is becoming increasingly important because of its higher reported sensitivity for the detection of primary tumor, regional lymph node involvement, and distant metastases than conventional imaging [14]. Moreover, FDG PET/CT, although not routinely used in the staging of patients with ACC, can significantly alter the initial stage in comparison with conventional diagnostic techniques in at least 20–25% of evaluated patients [15–18] and may be helpful in the target volume delineation in patients scheduled for radiation therapy with curative intent [19].

Sentinel lymph node biopsy procedure has proved to be a more accurate method than clinical or radiological techniques in staging the disease of patients with ACC, with detection rates up to 95–100% in the inguinal region [20, 21]. Conversely, the diagnostic performance of PET/CT using FDG in evaluating locoregional (inguinal, pelvic) lymph node involvement from ACC is unclear: several studies have been published over the years on this topic, with conflicting results; moreover, a meta-analysis of published studies was lacking. The aim of our study is to systematically review and meta-analyze published data about the diagnostic performance of PET/CT using FDG in detecting locoregional lymph node involvement in patients with biopsy-proven ACC, in order to provide evidence-based data in this setting.

## 2. Materials and Methods

### 2.1. Search Strategy

A comprehensive computer literature search of PubMed/MEDLINE, Scopus, and Embase databases was carried out to find relevant published articles concerning the diagnostic performance of FDG-PET in detecting locoregional nodal involvement in patients with biopsy-proven ACC. We used a search algorithm based on a combination of the terms “(anus OR anal) AND (PET OR positron emission tomography).” No language restriction was used. The search was performed from inception to July 10, 2013. To expand our search, references of the retrieved articles were also screened for additional studies.

### 2.2. Study Selection

Studies or subsets in studies investigating the role of positron emission tomography/computed tomography using FDG-PET in detecting locoregional nodal involvement in patients with biopsy-proven ACC were eligible for inclusion. Case reports, small case series, review articles, letters, editorials, and conference proceedings were excluded.

The following inclusion criteria were applied to select studies for this meta-analysis:

1. FDG-PET performed in patients with biopsy-proven ACC,
2. a sample size of at least ten patients with ACC who performed FDG-PET in the course of their diagnostic workup,
3. sufficient data to reassess sensitivity and specificity of FDG-PET in patients with ACC, and
4. no data overlap (when possible duplicate studies were found, only the most complete article was included).

Two researchers (C. Caldarella and S. Annunziata) independently reviewed titles and abstracts of the retrieved articles, applying the above-mentioned selection criteria. Articles were rejected if clearly ineligible. The same two researchers then independently evaluated the full-text version of the included articles to determine their eligibility for inclusion.

### 2.3. Data Extraction

Information about basic study (authors, journal, year of publication, and country of origin), study design (prospective or retrospective), patients' characteristics (number of patients with anal canal cancer performing FDG-PET, mean age, and gender), technical aspects (injected dose of FDG and acquisition modality), and reference standard results (benign versus malignant histology) was collected.

Each study was analyzed to retrieve the number of true-positive (TP), true-negative (TN), false-positive (FP), and false-negative (FN) findings of FDG-PET in patients with ACC, according to the reference standard. Only studies providing such complete information were finally included in the meta-analysis.

### 2.4. Quality Assessment

Two independent reviewers (R. Sadeghi and N. Ayati) evaluated the methodology of the selected studies using the “2011 Oxford Center for Evidence-Based Medicine Level of Evidence” (available at http://www.cebm.net/index.aspx?o=5653) assessment tool for diagnostic performance evaluation. For each included paper, this tool takes into account several parameters: spectrum of the studied cases, recruitment of patients (consecutive/not consecutive), reference standard, ascertainment of the gold standard regardless of the index test results (Yes/No), blind comparison of the index test and reference standard (Yes/No), enough explanation of the index test to ensure reproducibility (Yes/No), and study design (prospective/retrospective). A level-of-evidence score is obtained, ranging from 1 to 5.

### 2.5. Statistical Analysis

At first, sensitivity and specificity of FDG-PET in ACC were obtained from the individual studies, on a per lesion-based analysis. We considered as positive a node with an increased uptake of FDG, according to the criteria reported by different authors. When a positive node was histologically confirmed as malignant, this was considered a TP lesion, whereas a histologically confirmed benign node was considered as a FP finding. We considered as negative a node with no uptake of FDG: when the node was histologically confirmed as malignant, this was considered a FN lesion, whereas a histologically confirmed benign node was considered as a TN finding.

Sensitivity was determined according to the following formula: TP/(TP + FN); specificity was determined according to this formula: TN/(TN + FP). Statistical pooling of the data was performed by means of a random effects model. Pooled data are presented with 95% confidence intervals (95% CI). Heterogeneity between studies was assessed by a *I* ^2^ statistic. A receiving operator characteristics (ROC) curve was obtained for selected studies and area under curve (AUC) was calculated to assess the overall accuracy of FDG-PET. Statistical analyses were performed using Meta-DiSc statistical software version 1.4 (available at: http://www.hrc.es/investigacion/metadisc_en.htm).

## 3. Results

### 3.1. Literature Search

**Figure 1:**
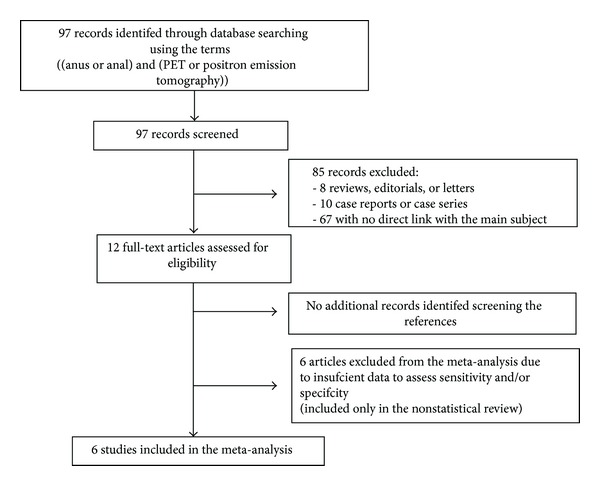
Flowchart of the search for the eligible studies on the diagnostic performance of FDG PET/CT in detecting locoregional node involvement in patients with ACC.

The comprehensive computer literature search from PubMed/MEDLINE, Embase, and Scopus databases revealed 97 articles (Figure 1). Most papers were excluded because they were not related to the main subject. Reviewing titles and abstracts, 12 articles were potentially eligible for inclusion applying the selection criteria mentioned above and were retrieved in full-text version; no additional studies were retrieved screening the references. Finally, 12 studies met all inclusion criteria and 6 were included in the meta-analysis [16, 23–26, 30]. Basic study characteristics and methodological aspects of the 12 retrieved studies are summarized in Tables 1 and 2. Raw data about true-positive, true-negative, false-positive, and false-negative findings in 6 studies are presented in Table 3.

### 3.2. Quality Assessment

Overall, the studies included in this meta-analysis have shown moderate methodological quality according to the “2011 Oxford Center for Evidence-Based Medicine Level of Evidence” assessment tool for diagnostic performance evaluation, with level-of-evidence score ranging from 3 to 4 for most papers. Use of poor/nonindependent reference standard was the main drawback of the included studies. Results of methodology assessment are summarized in Tables 4 and 5.

### 3.3. Literature Data Discussion

In this review, seven retrospective studies about the diagnostic performance of PET/CT using FDG in detecting locoregional lymph node involvement in patients with biopsy-proven ACC have been included.

Cotter et al. [16] evaluated the utility of FDG-PET as a further nonsurgical option in the staging of carcinoma of the anus, with specific attention to the role of FDG-PET in identification of inguinal lymph node metastases. Authors concluded that FDG-PET detects substantially more abnormal inguinal lymph nodes than detected by clinical examination or CT. Nguyen et al. [18] have assessed the value of FDG-PET in the pretreatment staging of ACC, as compared with the standard clinical assessment with using CT: staging of ACC could be improved by PET scanning by identifying nodal and distant disease involvement thus resulting in upstaging in up to one-fifth of the cases. Iagaru et al. [22] have studied a retrospective case series of patients diagnosed and treated for anal squamous cell carcinoma (ASCC). This group analyzed that FDG PET/CT could be an effective diagnostic tool in the imaging of ASCC by providing reliable information regarding the staging and management of this disease and the need for surgical biopsy. In the study by Mai et al. [23], the influence of N stage as defined by FDG-PET on patients' outcome was analyzed: the authors concluded that the reduction of the irradiation dose to CT-enlarged but PET-negative inguinal lymph nodes in ACC seems not to result in an increase of failure rates. Sveistrup et al. [26] have determined retrospectively the role of PET/CT in the staging of ACC and they have defined the influence of PET/CT on the initial staging and treatment plan proposed by the three-dimensional transanal ultrasound (TAUS): PET/CT seemed to be important for the N2/3 stage and M stage ACCs, as well as for the detection of eventual synchronous neoplasms. Bhuva et al. [28] have assessed the usefulness of PET/CT in addition to standard imaging and evaluated its impact on staging and management of ACC: this study showed that PET/CT findings alter the clinical staging in a significant amount of patients. Wells and Fox [29] have evaluated the role of FDG-PET in the current multidisciplinary management of ACC: PET/CT is particularly useful in further characterization of MRI findings of uncertain significance, thus allowing a more sensitive detection of recurrence but also avoiding unnecessary biopsy when PET/CT negative findings occur.

### 3.4. Five Prospective Studies Have Been Reviewed

De Winton et al. [15] have determined the effect of FDG-PET on the nodal staging, radiotherapy planning, and prognosis prediction in patients with primary ACC: the authors showed a change in nodal stage and a subsequent extent of radiation fields when PET was added to conventional imaging tools in a significant proportion of patients. In 2012, Mistrangelo et al. [17] have evaluated the role of PET/CT in the staging and followup of patients affected by ACC: although PET-CT proved to be useful in the initial staging of perirectal/pelvic or inguinal lymph nodes, currently inguinal lymph nodes are staged with much greater sensitivity by sentinel node biopsy. Engledow et al. [25] have investigated pretreatment staging in ACC by using PET/CT and whether this tool could alter the stage and management through the detection of local or distant disease: PET/CT is recommended in pretreatment staging of ACC, but the exact timing of posttreatment PET/CT for response evaluation remains to be determined. Finally, in 2010 Mistrangelo et al. [24] have compared FDG-PET/CT findings with the results of biopsy of the inguinal sentinel lymph node, to determine whether PET-CT could upstage the local disease: FDG-PET specificity and positive predictive values were not encouraging (83% and 43%, resp.). Therefore, sentinel node biopsy of inguinal lymph nodes should be considered as the technique of choice in this setting. In 2011, Vercellino et al. [27] evaluated the diagnostic performance of FDG-PET/CT for staging and monitoring response in ACC: the authors concluded that PET/CT could be useful in the diagnosis of recurrence or in a restaging setting, especially in cases when a salvage surgery is scheduled.

### 3.5. Pooled Diagnostic Performance

**Figure 2:**
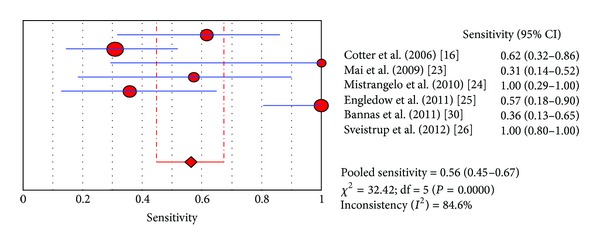
Plot of individual studies and pooled sensitivity of FDG PET/CT in detecting locoregional node involvement in patients with ACC, including 95% confidence interval. The size of the circles indicates the weight of each study.

**Figure 3:**
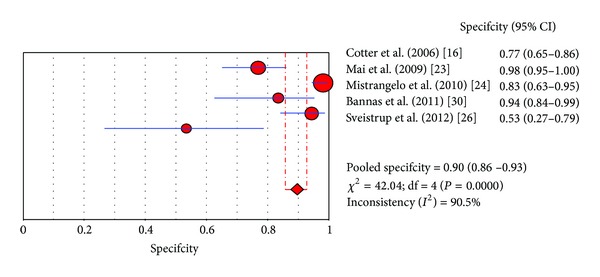
Plot of individual studies and pooled specificity of FDG PET/CT in detecting locoregional node involvement in patients with ACC on a per lesion-based analysis, including 95% confidence interval. The size of the circles indicates the weight of each study.

**Figure 4:**
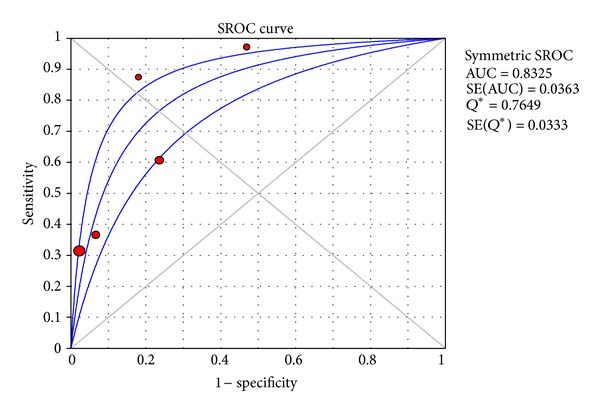
Summary SROC curve of diagnostic accuracy of FDG PET/CT in detecting locoregional node involvement in patients with ACC on a per lesion-based analysis, including 95% confidence interval.

The diagnostic performance values of FDG-PET/CT in the studies included in this meta-analysis are presented in Figures 2, 3, and 4. Six studies allowed assessing pooled sensitivity; five studies were used for pooled specificity. Sensitivity and specificity values of FDG-PET/CT on a per lesion-based analysis ranged from 31 to 100% and from 53 to 98%, with pooled estimates of 56% (95% CI: 45–67%) and 90% (95% CI: 86–93%), respectively. The included studies showed statistical heterogeneity in their estimate of sensitivity (*I* ^2^: 84.6%) and specificity (*I* ^2^: 90.5%). The area under the ROC curve was 0.83.

## 4. Discussion

ACC is a not common neoplasm, with an incidence of about 1–3 cases per 100,000 and accounting for about 2% of gastrointestinal malignancies [1–3]. ACC incidence rates are significantly higher in HIV-infected men and women than in HIV-uninfected ones, especially when considering men with homosexual habits [4–6].

To date, MRI performed is a good tool to investigate the anal region for both primary tumour and pelvic node involvement [9–12]. CT is a reliable alternative in detecting the primary lesion in patients with contraindications to MRI and allows detection metastases in other organs [13].

FDG PET/CT is not routinely used in the staging of patients with ACC but can significantly alter the initial stage in comparison with conventional diagnostic techniques in at least 20–25% of evaluated patients [15–18]. It is also used in the target volume delineation in patients scheduled for radiation therapy with curative intent [19].

Our meta-analysis aimed to assess the diagnostic performance of PET/CT using FDG in detecting locoregional lymph node involvement in patients with biopsy-proven ACC, in order to provide evidence-based data in this setting. Sensitivity and specificity values of FDG-PET/CT on a per patient-based analysis were of 56% (95% CI: 45–67%) and 90% (95% CI: 86–93%), respectively. False-negative findings reduce sensitivity, probably caused by high uptake of primary anal tumour and small size of node lesions. Although specificity was good, false-positive findings occur, especially when the extent of inguinal involvement is assessed, as inflammatory disease of pelvic region or lower extremities should be taken into account.

*Limits of the Study.* A systematic review process was adopted in ascertaining studies: to avoid selection bias, we adopted rigid inclusion criteria. Moreover, a methodological quality evaluation according to the “2011 Oxford Center for Evidence-Based Medicine Level of Evidence” has been performed: the index test and the reference standard were often interpreted without blinding, therefore limiting the methodological quality of the included studies.

Heterogeneity between studies may represent a potential source of bias; the included studies were statistically heterogeneous in their estimates of sensitivity and specificity. Since systematic reviews bring together studies that are different both clinically and methodologically, heterogeneity in their results should be expected. For example, heterogeneity is likely to arise through diversity in technical aspects, sample size, study quality, and inclusion criteria.

Publication bias is a major concern in all forms of pooled analyses since studies reporting significant findings are more likely to be published than those reporting nonsignificant results. Indeed, it is not unusual for small-sized early studies to report a positive relationship that subsequent larger studies fail to replicate. We cannot exclude a publication bias in our analysis, but we tried to minimize it by selecting only articles that included at least ten patients who performed FDG-PET with biopsy-proven anal canal cancer.

## 5. Conclusions

Our meta-analysis demonstrates that FDG-PET is a specific diagnostic tool in detecting locoregional lymph node involvement in patients with biopsy-proven ACC. Low sensitivity is a major concern, especially when considering small size lymph node involvement; however, a higher sensitivity could be reached by combining FDG-PET with MRI scan in the diagnostic management of patients with ACC, to reduce the risk of false negative results. Upcoming hybrid PET-MRI hybrid tomographs are expected to improve the diagnostic performance of FDG-PET in these patients.

## Figures and Tables

**Table 1:** Basic characteristics of 12 selected studies.

Authors	Year	Country	Type of study	Number of patients who performed PET/CT	Gender (% male)	Mean age
Cotter et al. [16]	2006	USA	Retrospective	41	44%	52
Nguyen et al. [18]	2008	Australia	Retrospective	50	38%	58
Iagaru et al. [22]	2009	USA	Retrospective	8	75%	44
Mai et al. [23]	2009	Germany	Retrospective	39	44%	59
De Winton et al. [15]	2009	UK	Prospective	61	44%	57
Mistrangelo et al. [24]	2010	Italy	Prospective	27	33%	58
Mistrangelo et al. [17]	2012	Italy	Prospective	53	36%	57
Engledow et al. [25]	2011	UK	Prospective	40	60%	57
Sveistrup et al. [26]	2012	Denmark	Retrospective	95	32%	58
Vercellino et al. [27]	2011	France	NR	58	30%	62
Bhuva et al. [28]	2012	UK	Retrospective	88	NR	NR
Wells and Fox [29]	2012	UK	Retrospective	44	NR	NR

NR: not reported.

**Table 2:** Methodological aspects of 12 selected studies.

Authors	Year	PET device	Mean injected activity (MBq)	Time between injection and acquisition	PET image analysis
Cotter et al. [16]	2006	PET/CT	Range 555–740	60	Visual
Nguyen et al. [18]	2008	PET or PET/CT	Range 370–400	60	Visual
Iagaru et al. [22]	2009	PET	NR	60	Visual and semiquantitative
Mai et al. [23]	2009	PET	355	60	Visual and semiquantitative
De Winton et al. [15]	2009	PET or PET/CT	Range 300–400	60	Visual
Mistrangelo et al. [24]	2010	PET/CT	Range 222–370	60	Visual
Mistrangelo et al. [17]	2012	PET/CT	NR	NR	Visual
Engledow et al. [25]	2011	PET/CT	375	60	Visual
Sveistrup et al. [26]	2012	PET/CT	400	60	Visual
Vercellino et al. [27]	2011	PET/CT	5/kg	60	Visual
Bhuva et al. [28]	2012	PET/CT	4.5/kg	60	Visual
Wells and [29]	2012	PET/CT	NR	NR	Visual and semiquantitative

NR: not reported.

**Table 3:** Number of true-positive, true-negative, false-negative, and false-positive results of 6 studies collected on a per lesion-based analysis.

Author	TP	TN	FN	FP
Cotter et al. [16]	8	53	5	16
Mai et al. [23]	8	154	18	3
Mistrangelo et al. [24]	3	20	0	4
Engledow et al. [25]	4	0	3	2
Bannas et al. [30]	5	49	9	3
Sveistrup et al. [26]	17	8	0	7

**Table 4:** Quality assessment according to 2011 Oxford Center for Evidence-Based Medicine Level of Evidence.

First author and publication year	Spectrum of the studied cases	Consecutive recruitment of the patients	Reference standard	Ascertainment of the gold standard regardless of the index test results	Blind comparison of the index test and reference standard	Enough explanation of the index test to ensure reproducibility	Prospective/ retrospective design	2011 Oxford Center for Evidence-Based Medicine Level of Evidence (http://www.cebm.net/ index.aspx?o=5653)
Bannas 2011 [30]	Patients with biopsy-proven carcinoma of the anus	Yes	Complete staging evaluation including physical examination, biopsy of the primary tumor, and contrast-enhanced (ce)-PET/CT. Final diagnosis was by a consensus report of PET/CT	No	Yes	Yes	Retrospective	4 (nonindependent reference standard)

Bhuva 2012 [28]	All patients undergoing radical treatment for anal cancer	No	Clinical examination, CT scans of the chest, abdomen and pelvis, MRI scans of the pelvis, and whole-body FDG-PET/CT scans	No	Yes	Yes	Retrospective	4 (nonindependent reference standard)

Cotter 2006 [16]	Patients with biopsy-proven carcinoma of the anus	Yes	Complete staging evaluation including physical examination, CT, and 2-FDG-PET/CT	Yes	No	Yes	Retrospective	4 (nonindependent reference standard)

De Winton 2009 [15]	Patients with primary anal canal cancer	Yes	Conventional imaging (including computed tomography (CT), magnetic resonance imaging, endoscopic ultrasound, and chest X-ray) and followup of the patients	Yes	Yes	Yes	Prospective	2

Engledow 2011 [25]	Patients with a histologically confirmed anal SCC	N/A	Computed tomography, magnetic resonance imaging, and examination under anaesthetic. Histology for suspicious lymph nodes	Yes	Yes	Yes	Prospective	3 (no information regarding consecutive patient recruitment)

Iagaru 2009 [22]	Patients with proven anal SCC	Yes	Conventional imaging and histology	Yes	N/A	Yes	Retrospective	3 (no information regarding blinding)

Krengli 2010 [19]	Patients with biopsy proven anal carcinoma	Yes	Conventional imaging (CT) and histology in FDG-positive cases	No	N/A	Yes	Prospective	3 (inconsistent application of gold standard)

Mai 2009 [23]	Histologically proven epidermoid anal cancer	Yes	Conventional imaging (CT)	Yes	Yes	Yes	Retrospective	4 (poor reference standard)

Mistrangelo 2010 [24]	Patients with anal cancer	N/A	Sentinel node mapping	Yes	N/A	Yes	N/A	4 (poor reference standard)

Mistrangelo 2012 [17]	Patients with anal cancer (either staging or restaging)	Yes	Digital rectal examination, anoscopy and biopsy of suspicious lesions, chest X-ray, rectal endosonography, endoscopy, CT or MRI, sentinel node mapping (for inguinal node staging), and anal biopsy (for detection of persistent disease)	No	N/A	Yes	N/A	4 (poor reference standard for staging) 3 (blinding, not reported for persistent disease detection)

Nguyen 2008 [18]	Patients with histopathologically confirmed epidermoid carcinoma	Yes (no for detection of recurrence)	For staging: complete history and full physical examination which included anorectal examination, palpation of the inguinofemoral nodes, anal biopsy and CT of the chest, abdomen, and pelvis/histology for detection of recurrence	Yes	No	Yes	Retrospective	4 (poor reference standard for staging) 3 (non-consecutive recruitment for detection of recurrence)

Sveistrup 2012 [26]	All patients with anal cancer	Yes	Transanal three-dimensional (3D) ultrasound and US of the inguinal regions/biopsy of the suspicious inguinal nodes	No	N/A	Yes	Retrospective	3 (inconsistently used reference standard)

Vercellino 2011 [27]	Patients with anal carcinoma (either staging or re-staging)	N/A	Histological confirmation if available otherwise followup for at least 6 months (physical and proctologic examinations and serial imaging studies such as CT, MRI, US, or subsequent FDG PET/CT examinations)	Yes	No	Yes	Prospective	3 (no information regarding consecutive patient recruitment)

Wells 2012 [29]	Patients with anal cancer (either staging or re-staging)	Yes	CT (thorax, abdomen, and pelvis) and pelvic MRI	Yes	N/A	Yes	Retrospective	4 (poor reference standard)

**Table 5:** Quality assessment according to 2011 Oxford Center for Evidence-Based Medicine Level of Evidence.

First author and publication year	Population of the patients included in the study	Duration of followup (mean or median)	Loss to followup	Main prognostic factor	Evaluated outcome	Objective/blind method for outcome evaluation	Adjustment for other confounding prognostic factors	2011 Oxford Center for Evidence-Based Medicine Level of Evidence (http://www.cebm.net/ index.aspx?o=5653)
Cotter 2006 [16]	Patients with biopsy-proved carcinoma of the anus	15.2 months	None	FDG uptake in the inguinal nodes	Overall or progression-free survivals/HR not reported	Not mentioned	No	4 (no information regarding method of recurrence evaluation)

De Winton 2009 [15]	Patients with anal cancer who were referred to a tertiary centre	2.6 years (median)	None	FDG PET staging (N stage only)	Overall survival and progression-free survival/HR not reported however survival curves are provided	Pathology, therapeutic response, imaging, clinical followup, and concordance between conventional imaging and PET	No	4 (inception cohort study without adjustment for confounding factors)

Mai 2009 [23]	Patients with histologically proven epidermoid anal cancer referred to a radiation oncology department	26 months (mean)	1/39 patients	FDG PET staging (N staging only)	Freedom from metastases/HR not reported but survival curve is provided	Not mentioned	No	4 (retrospective cohort study with no information regarding the tests to evaluate outcome)

Nguyen 2008 [18]	All patients with histopathologically confirmed epidermoid carcinoma of the anus referred to a PET center	25 months (median)	None	FDG PET metabolic response	Progression-free survival/HR not reported and survival curve not provided	Clinical examination, PDF PET imaging, and histological confirmation	No	4 (retrospective cohort study without adjusting for confounding factors)
